# Downregulation of miR-183 inhibits apoptosis and enhances the invasive potential of endometrial stromal cells in endometriosis

**DOI:** 10.3892/ijmm.2013.1536

**Published:** 2013-10-25

**Authors:** XIAO-YAN SHI, LIN GU, JIE CHEN, XI-RONG GUO, YING-LI SHI

**Affiliations:** State Key Laboratory of Reproductive Medicine, Department of Gynecology, Nanjing Maternity and Child Health Care Hospital Affiliated to Nanjing Medical University, Nanjing, Jiangsu 210029, P.R. China

**Keywords:** endometriosis, microRNA-183, endometrial stromal cell, pathogenesis

## Abstract

Endometriosis is a common gynecological disease, yet its pathogenesis remains poorly understood. Recent studies have demonstrated that the aberrant expression of certain microRNAs (miRNAs) may correlate with the development and progression of endometriosis. In this study, we profiled several differentially expressed miRNAs in the normal, eutopic and ectopic endometrium by miRNA microarray screening analysis, among which, miR-183 was found to be downregulated in the ectopic and eutopic tissues, and the result was further confirmed by real-time PCR (qPCR). Functional analysis indicated that miR-183 plays a promotional role in endometrial stromal cell (ESC) apoptosis and has a negative regulatory impact on the invasive ability of cells, although it has no effect on ESC proliferation. Ovarian steroids (17β-estradiol and progesterone) and inflammatory factors (tumor necrosis factor-α and interleukin-6) decreased the expression of miR-183 in the ESCs. This regulatory function may further manifest the growth and invasive potential of ESCs by altering the expression of miR-183. These findings suggest that the downregulation of miR-183 expression is involved in the development and progression of endometriosis.

## Introduction

Endometriosis is a common gynecological disease associated with severe pelvic pain, affecting 6–10% of women in their reproductive years and 20–50% of infertile women (1).

The development of novel methods for the diagnosis or treatment of endometriosis is hampered due to the lack of knowledge regarding the etiology, pathogenesis and natural progression of endometriosis. Although there have been several studies published on this disease, its causative mechanisms have yet to be identified (2). Retrograde menstruation is still considered the most prominent cause among the various theories put forward to explain the pathogenesis of endometriosis. The majority of women have retrograde menstruation; however, only 10–15% of women suffer from endometriosis (3), suggesting that other factors are involved in the development of endometriosis.

microRNAs (miRNAs) are small non-coding regulatory RNAs that regulate the translation of mRNAs by inhibiting ribosomal functions, decapping the 50 Cap structure, deadenylating the poly(A) tail and degrading the target mRNA (4). miRNAs are able to regulate the expression of hundreds of target mRNAs simultaneously, thus controlling a variety of cellular functions, including cell proliferation, stem cell maintenance and differentiation (5). Aberrant miRNA expression is associated with human diseases, such as gynecological diseases, cancer, inflammatory diseases and cardiovascular disorders. Emerging data indicate a different molecular environment and altered miRNA expression in the pathological endometrium, in contrast to the normal endometrium (6–9).

In this study, a systematic comparison of the miRNAs in the ectopic, eutopic and normal endometrium was conducted, leading to the identification of miR-183, which has a regulatory impact on the development of endometriosis.

## Materials and methods

### Tissue acquisition

All the endometriotic, eutopic and normal endometrial tissues were obtained at Nanjing Maternity and Child Health Care Hospital Affiliated to Nanjing Medical University, Nanjing, China by laparoscopy and uterine curettage from patients with or without endometriosis. None of the patients had received pre-operative hormonal therapy, and all the samples were histologically confirmed. All the samples were from the proliferative phase of the menstrual cycle from pre-menopausal women. The phase of the menstrual cycle was determined by pre-operative history and a histological evaluation of the endometrium. The normal samples were obtained from patients with an average age of 38.9±4.8 years and the endometriosis samples were otbained from patients with an average age of 36.6±5.1 years. All patients provided written informed consent prior to participating in this study. This study was approved by the hospital ethics committee. Each sample was divided and used for mRNA extraction, as well as cell isolation.

### miRNA microarray

Microarrays were performed by utilizing the miRCURY LNA™ microRNA Array (v. 14.0; Exiqon, Vedbaek, Denmark). All procedures were carried out according to the manufacturer’s instructions. Following RNA measurement on the NanoDrop instrument, the samples were labeled using the miRCURY™ Hy5™/Hy3™ Power labeling kit (Exiqon) and hybridized on the miRCURY LNA™ Array (v. 14.0), which contains >1,700 capture probes covering all miRNAs listed in miRBase v. 14.0. Following the labeling procedure, the Hy3™-labeled samples and a Hy5™-labeled reference RNA sample were mixed pairwise and hybridized using the miRCURY LNA™ Array v. 14.0 (Exiqon). The hybridization and subsequent wash steps were performed according to the miRCURY LNA™ Array manual. The microarray slides were scanned using the Axon GenePix 4000B microarray scanner (MDS Analytical Technologies, Silicon Valley, CA, USA) and image analysis was carried out using GenePix pro v. 6.0 software (MDS Analytical Technologies). The normalized data were analyzed using the locally weighted scatter plot smoothing (lowess) regression algorithm [TIGR Microarray Data Analysis System (MIDAS)]. Following normalization, the differentially expressed miRNAs were identified through fold change filtering (fold change >2.0). Hierarchical clustering was performed using MEV software (v. 4.6, TIGR).

### Cell culture and treatment

The endometrial stromal cells (ESCs) from women with or without endometriosis were cultured according to a previously described method (10). Following serum starvation for 12 h, the ESCs (1×10^5^ cells/well) were treated with 17β-estradiol (E2) (10^−8^ mol/l), progesterone (P) (10^−8^ mol/l), E2 (10^−8^ mol/l) + P (10^−8^ mol/l), interleukin-6 (IL-6) (10 ng/ml) or tumor necrosis factor-α (TNF-α) (10 ng/ml) for 24 h; vehicle controls were also used (treated with ethanol, 17β-estradiol solution).

### Fluorescence-based real-time PCR (qPCR)

Total RNA was isolated using TRIzol reagent (Takara, Otsu, Shiga, Japan) and cDNA was synthesized using the SYBR^®^ PrimeScript™ RT-PCR kit (Takara) on the ABI PRISM 500 Sequence Detection System (Applied Biosystems, Foster City, CA, USA) according to the manufacturer’s instructions. The housekeeping gene encoding Hsa-U6 small nuclear RNA (snRNA) was used for normalization. The primers used were as follows: 5′-CGCG CGTGAATTACCGAAG-3′ (forward) and 5′-GTGCAGGG TCCGAGGT-3′ (reverse) for miR-183-3p; 5′-CGCGCTAT GGCACTGGTAG-3′ (forward) and 5′-GTGCAGGGTCC GAGGT-3′ (reverse) for miR-183-5p; and 5′-GCGCGTCGTG AAGCGTTC-3′ (forward) and 5′-GTGCAGGGTCCGAGGT-3′ (reverse) for Hsa-U6 snRNA. The conditions for qPCR were as follows: 95°C for 5 min; 45 cycles of 95°C for 15 sec followed by 60°C for 30 sec and 72°C for 30 sec; 95°C for 55 sec, 50°C for 2 min, 95°C for 10 min, 40 cycles of 95°C for 15 sec and 60°C for 1 min. qPCR was carried out using SYBR-Green JumpStart Taq ReadyMix (Sigma-Aldrich, St. Louis, MO, USA) and the 7300 Real-Time PCR Detection System (ABI). The results were analyzed using the comparative threshold cycle (Ct) method.

### miR-183 lentivirus construction and transduction

The precursor of the miRNA, hsa-miR-183 (GenBank accession no. MIMAT0000261), and the inhibitor of hsa-miR-183-5p lentivirus gene transfer vector encoding green fluorescent protein (GFP) were constructed by Genechem Co., Ltd. (Shanghai, China).

The RNA primers used were: 5′-GAGGATCCCCGGG TACCAAGGGAGTGGGCAGGCTA-3′ and 5′-ATAAGCTTG ATATCGTCCCTGCACCCTTGGAAGCA-3′, and were confirmed by sequencing. The recombinant lentivirus of overexpressed miR-183 (miR-183-lentivirus) and the control lentivirus (GFP-lentivirus) were prepared and titered to 5.0 E+8 TU/ml (transfection unit).

The sequence of the inhibitor of hsa-miR-183-5p was TATGGCACTGGTAGAATTCACT, and confirmed by sequencing. The recombinant lentivirus of miR-183-5p inhibitor (In-miR-183-lentivirus) and the control lentivirus (GFP-lentivirus) were prepared and titered to 4.0 E+8 TU/ml (transfection unit).

ESCs from women without endometriosis were plated in 6-well plates (5×10^4^ cells/well) overnight. The lentiviruses were diluted in 0.2 ml complete medium containing polybrene (8 mg/ml) and added to the cells for 12 h of incubation at 37°C, followed by incubation in 0.3 ml of freshly prepared polybrene-DMEM for another 24 h, which was replaced with fresh DMEM and the cells were cultured for 3 days. The lentivirus transduction efficiency of the ESCs was determined by the detection of GFP signals by fluorescence microscopy at 72 h after transduction. The miR-183 expression in the stably transduced ESCs was measured by qPCR. The ESCs transfected with miR-183-lentivirus, In-miR-183-lentivirus and GFP-lentivirus were kept for further functional analysis.

### Measurement of apoptotic cell death by flow cytometry

Apoptosis assay was performed according to the operation manual provided with the BD Annexin V Staining kit (BD Biosciences, Franklin Lakes, NJ, USA). Briefly, the ESCs were infected with lentivirus for 5 days under serum-free conditions, the cells were trypsinized and collected by centrifugation at 1,500 rpm for 5 min, washed once with phosphate-buffered saline (PBS) at 4°C and then resuspend in 1X binding buffer at a concentration of 1×10^6^ cells/ml. Subsequently, 100 μl of the solution (1×10^5^ cells) were transferred to a 5-ml culture tube. Annexin V and PI (5 μl/test tube) were then added and the cells were gently mixed and incubated for 15 min at room temperature in the dark. This was followed by the addition of 400 μl of 1X binding buffer to each tube. The cells were then analyzed by flow cytometry as soon as possible (within 1 h).

### Measurement of cell viability by MTT

The ESCs (2.0×10^3^ cells/well) were seeded into 96-well plates (Corning Costar, Corning, NY, USA) in DMEM supplemented with 10% fetal bovine serum (FBS) and incubated for 1–5 days. Following incubation, 10 μl of MTT (Sigma-Aldrich) solution (5 mg/ml in ddH_2_O) were added to each well. The plates were incubated for a further 4 h at 37°C. Intracellular formazan crystals were dissolved by the addition of 100 μl of DMSO to each well. Cell proliferation was evaluated by measuring the absorbance at 490 nm.

### Invasion (Matrigel) chamber assay

The ESCs (2.5×10^4^) were seeded on a cell culture Transwell insert coated with extracellular matrix (ECM) (8-mm pore size, 24-well format; Corning Costar) in 2% FBS medium, and complete medium (10% FBS) was added to the lower chamber. To determine the amount of invading cells, the cells were incubated for 24 h and then removed from the upper chamber using a cotton swab. The invaded cells on the underside of the insert were fixed with methanol (2 min). Once fixed, the cells were stained with crystal violet for 2 min and rinsed with PBS. The undersides of the membrane were then photographed to compare the number of invaded cells per insert. The transmigrated cells were counted under a light microscope. The invaded cells were scored by counting 10 random high-power fields per filter. The counting accuracy was guaranteed by optical density (OD)570 quantification of the methanol-solubilized dye.

### Statistical analysis

Data represent the means ± SEM of at least 3 independent experiments. The difference between 2 means was examined by the Student’s t-test, while one-way ANOVA was employed to compare 3 or more groups. A value of P<0.05 was considered to indicate a statistically significant difference.

## Results

### miRNA expression profile

**Figure 1:**
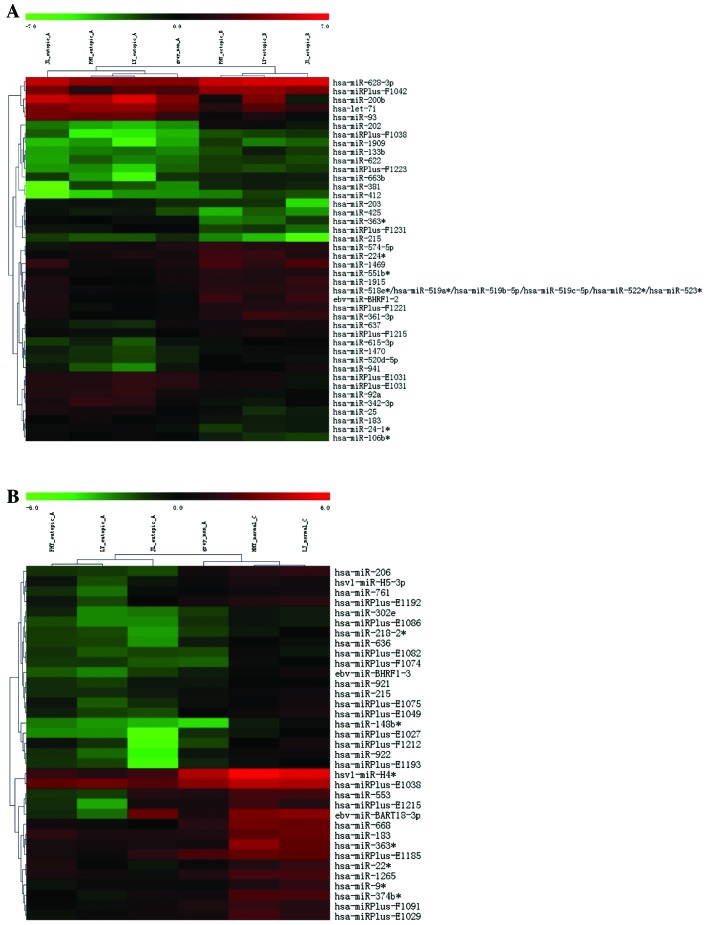
Hierarchical clustering of the 3 groups (ectopic endometrium, eutopic endometrium and normal endometrium). (A) Ectopic vs. eutopic endometrium, and (B) eutopic vs. normal endometrium. Distinguishable miRNA expression profiling is observed. Red indicates high relative expression, green indicates low relative expression and black represents zero.

Following the selection of miRNAs by fold change filtering (fold change >2.0), we found that there were 26 upregulated miRNAs and 19 downregulated miRNAs in the ectopic endometrium compared with the eutopic endometrium. Compared with the normal endometrium, 36 downregulated with no upregulated miRNAs were found in the eutopic endometrium (Table I). Among these differentially expressed miRNAs, miR-183, miR-215 and miR-363 were found downregulated both in the ectopic and eutopic tissues, from which we selected miR-183 as the target miRNA as it was the most significantly downregulated (Fig. 1).

### Quantification of miR-183 expression

**Figure 2:**
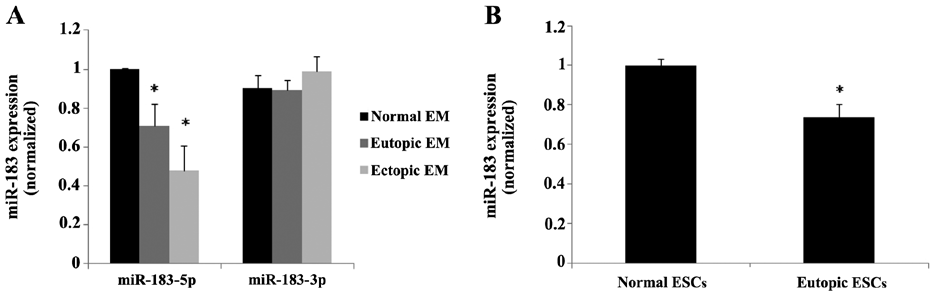
Validation of miR-183 expression by qPCR. (A) Ectopic and eutopic endometrium from women with endometriosis (n=20) and normal control endometrium from women without endometriosis (n=20). (B) Endometrial stromal cells (ESCs) from women with endometriosis and endometriosis-free women. miR-183 expression data are presented as the fold change compared with the normal group (^*^p<0.05). EM, endometrium.

The statistical data indicated that miR-183-5p was significantly downregulated in the ectopic and eutopic endometrial tissues from patients with endometriosis compared with those with a normal endometrium (P=0.011 and P=0.0002, respectively), whereas miR-183-3p did not show statistically significant differences in expression (Fig. 2A). Since the adhesion of endometrial cells to the peritoneal lining is a crucial step in the early stages of endometriosis, which is dependent on stromal cells, we further detected the expression of miR-183 in the ESCs isolated from the ectopic, eutopic and normal endometrium. The results of cell analysis conformed with the tissue samples (P<0.05) (Fig. 2B).

### Transfection efficiency of recombinant lentivirus

**Figure 3:**
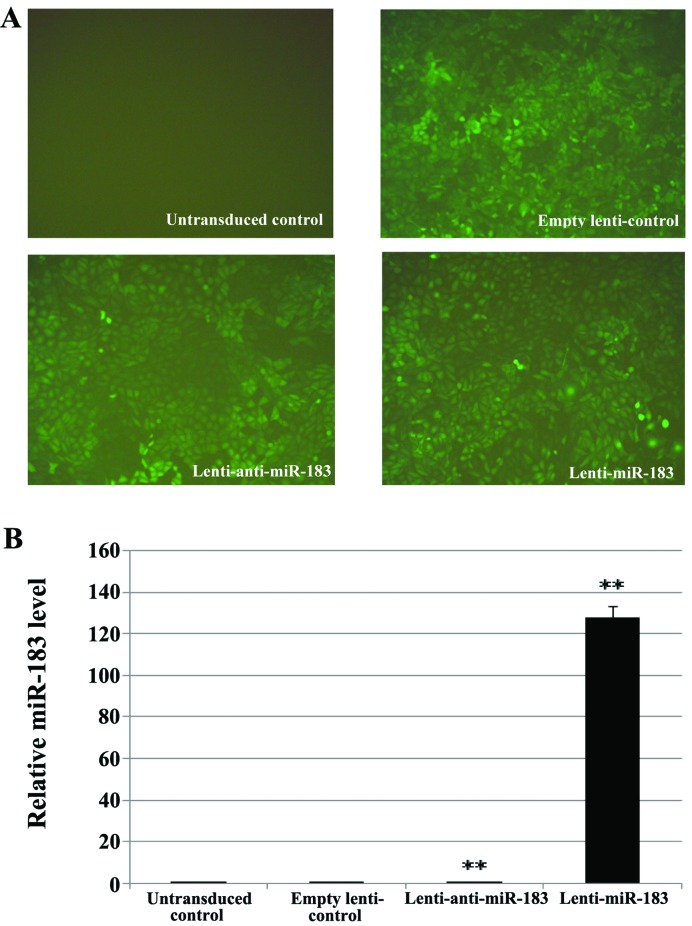
Stable lentiviral transduction of anti-miR-183-5p and miR-183 plasmid into endometrial stromal cells (ESCs). (A) Transduction efficiency was determined by fluorescence microscopy following transduction with recombinant lentivirus. (B) The expression levels of endogenous miR-183 were determined by qPCR. ^**^P<0.01, significantly different from both the untransduced and empty lentivirus control groups.

To further investigate the roles of miR-183 in ESCs, we prepared a lentiviral construct for the overexpression of miR-183 and anti-miR-183. Fig. 3A shows that the majority of the ESCs (>80%) expressed GFP and exhibited a morphology that was similar to that of naive ESCs. qPCR revealed that the transduction of ESCs with miR-183-lentivirus increased miR-183 expression by 127-fold, whereas transfection with In-miR-183-lentivirus increased it by only 0.15-fold (Fig. 3B).

### miR-183 decreases the invasive ability of ESCs

**Figure 4:**
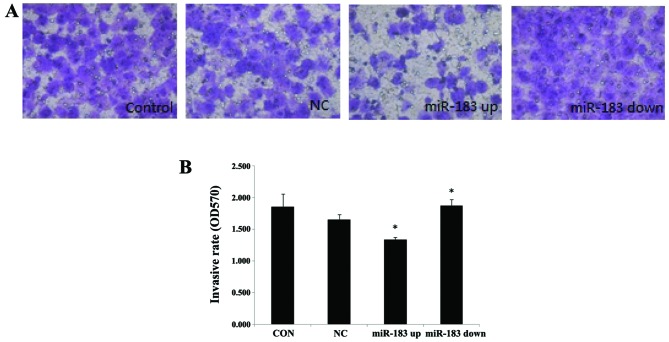
Effect of overexpression and downregulation of miR-183 on the invasive potential of endometrial stromal cells (ESCs). Matrigel invasion data when ESCs infected with miR-183-lentivirus, In-miR-183-lentivirus (miR-183 inhibitor) and green fluorescent protein (GFP)-lentivirus (control) in upper well were incubated in 2% FCS medium and lower well was filled with 10% FCS medium. After 24 h, the number of cells that invaded through Matrigel was counted in at least 10 fields/well. (A) Representative images reveal the ESCs that invaded through Matrigel. The ESCs in which miR-183 expression was downregulated, had a greater invasive potential. (B) Counting accuracy was guaranteed by optical density quantification. CON, control; NC, negative control.

To illustrate the function of miR-183 in the development of endometriosis, we first examined the effects of miR-183 overexpression and knockdown in ESCs using an invasion chamber coated with ECM-Matrigel and found that a significantly greater number of control ESCs (1.652±0.083) compared with miR-183-overexpressing ESCs (1.335±0.035) had passed through the matrix (P<0.05). By contrast, the ESCs in which miR-183 expression was downregulated (1.874±0.099) had greater invasive potential compared with the control cells (P<0.05) (Fig. 4).

### miR-183 induces the apoptosis of ESCs but has no effect on cell proliferation

**Figure 5:**
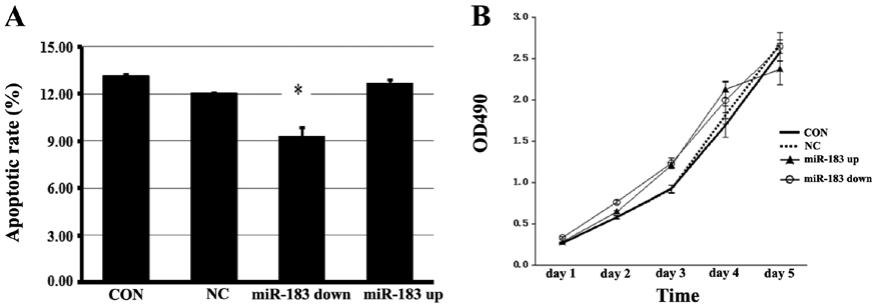
Effect of overexpression and downregulation of miR-183 on growth of endometrial stromal cells (ESCs). (A) Cell apoptosis was measured by Annexin V-FIFC. (B) Growth curves of ESCs transfected with miR-183-lentivirus, In-miR-183-lentivirus (miR-183 inhibitor) and green fluorescent protein (GFP)-lentivirus (control). Cell proliferation was measured by MTT assay. Data are presented as the means ± SD, ^*^p<0.05 and ^**^p<0.001. CON, control; NC, negative control.

The results of Annexin V assay demonstrated that the overexpression of miR-183 significantly induced the apoptosis of ESCs (P<0.05), and that the apoptotic rate was decreased when miR-183 was inhibited (P<0.05) (Fig. 5A). miR-183 did not significantly alter the cell proliferation rate (Fig. 5B).

### Ovarian steroids and inflammatory factors inhibit the expression of miR-183

**Figure 6:**
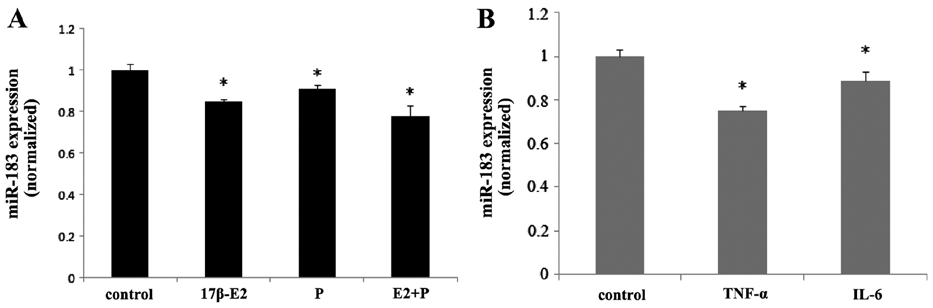
Effects of (A) ovarian steroids and (B) inflammatory factors on miR-183 expression in endometrial stromal cells (ESCs) assessed by qPCR. miR-183 expression data are presented as the foldachange relative to the normal group, ^*^p<0.05. 17β-E2, 17β-estradiol; P, progesterone; TNF, tumor necrosis factor; IL, interleukin.

qPCR demonstrated that the treatment of ESCs with ovarian steroids and inflammatory factors regulated the expression of miR-183. Both 17β-estradiol and progesterone inhibited the expression of miR-183 in ESCs (P<0.05), and co-treatment with these steroids induced synergistic effects (P<0.05) (Fig. 6A). Treatment of the ESCs with TNF-α and IL-6 also inhibited the expression of miR-183 (P<0.05) (Fig. 6B).

## Discussion

Endometriosis is a prevalent gynecological disease characterized by the growth of endometriotic tissue outside the uterine cavity. miRNAs are naturally occurring post-transcriptional regulatory molecules that potentially play a role in endometriotic lesion development (11). In recent years, emerging evidence suggests that the dysregulation of miRNA expression is involved in endometriosis. Previously, Ohlsson Teague *et al*(12) screened miRNA expression by microarray analysis in paired ectopic and eutopic endometrial tissues and identified 14 upregulated (miR-145, miR-143, miR-99a, miR-99b, miR-126, miR-100, miR-125b, miR-150, miR-125a, miR-223, miR-194, miR-365, miR-29c and miR-1) and 8 downregulated (miR-200a, miR-141, miR-200b, miR-142-3p, miR-424, miR-34c, miR-20a and miR-196b) miRNAs. More recently, Hawkins *et al*(13) also found 10 upregulated (miR-202, 193a-3p, 29c, 708, 509-3-5p, 574-3p, 193a-5p, 485-3p, 100 and 720) and 12 downregulated (miR-504, 141, 429, 203, 10a, 200b, 873, 200c, 200a, 449b, 375 and 34c-5p) miRNAs in endometriomas compared with the normal endometrium using next-generation sequencing technology. Many of the identified miRNAs, such as miR-199a, miR-126 and miR-10b were subsequently investigated in recent studies (14–16), further indicating that miRNAs play a role in the development of endometriosis.

Published studies have identified differentially expressed miRNAs in endometriotic tissues. However, to the best of our knowledge, the differences in miRNA expression in endometrial tissue from women with or without endometriosis have not yet been validated. It is well known that eutopic endometrial cells may function differently in women with endometriosis compared with a normal endometrium in disease-free women. These cells have more chances of survival outside the uterine cavity, which lead to the development of well-documented changes at the peritoneum and other ectopic sites (17). A heritable or acquired molecular aberration within the endometrium may have an impact on selective survival advantage to refluxed endometrial tissue in women predisposed to the development of endometriosis. The identification of molecular differences in the eutopic endometrium of women with endometriosis is an important step toward understanding the pathogenesis of this condition and toward developing effective strategies for the treatment of associated infertility and pain (17). In this study, we detected the miRNA expression profiles in endometrial tissue from endometriosis-free women, as well as in tissue from eutopic endometrium from women with surgically confirmed endometriosis and the tissue from ectopic endometrium. The results revealed 26 upregulated miRNAs and 19 downregulated miRNAs in the ectopic endometrium compared with the eutopic endometrium. Compared with the normal endometrium, 36 downregulated with no upregulated miRNAs were found in the eutopic endometrium. Among these differentially expressed miRNAs, miR-183, miR-215 and miR-363 were found to be downregulated in both the ectopic and eutopic tissues, from which we selected miR-183 as the target miRNA as it was the most significantly downregulated. The differential expression of miR-183 was further confirmed by qPCR. However, little is known about the role of miR-183 in the development of endometriosis.

The adhesion of endometrial cells to the peritoneal lining is a crucial step during the early stages of endometriosis, which is dependent on stromal cells, as no cell adhesion in the endometrial epithelium is identified during the first 24–48 h (18). In our study, we therefore selected ESCs as the target cells. The results of qPCR further confirmed that miR-183 expression in the eutopic endometrium was higher than that in the control group.

miR-183 is an miRNA involved in the regulation of cell growth, cell differentiation, apoptosis, cell motility, cell adhesion and cell invasion (19–21). It has been reported that miR-183 is upregulated in colorectal cancer, prostate cancer and hepatocellular carcinomas, while it is downregulated in ovarian cancer, breast cancer stem cells and osteosarcomas (5,20,22–25), suggesting that its roles vary depending on the cellular context.

In this study, we used ESCs as an *in vitro* model of endometriosis to examine the functional impact of miR-183 overexpression and inhibition on endometriotic cell behavior. Functional analysis indicated that miR-183 plays a promotional role in ESC apoptosis and exhibits a negative regulatory impact on the invasive ability of the cells; however, it has no effect on ESC proliferation. The cause of endometriosis has been attributed to the attachment and invasion of the retrograded endometrial fragments into the peritoneum, where they establish a blood supply, triggering a suboptimal immune response that does not adequately clear the implants, which induces their continued survival and growth. This effect of miR-183 can be expected *in vivo* to enhance the implantation and establishment of the ectopic lesion.

Hormonal alterations may influence the ability of endometrial cells to proliferate, attach to the mesothelium and evade immune-mediated clearance. In addition to the concept of endometriosis as an estrogen-dependent disorder, there is increasing evidence to suggest that an incomplete transition of the endometrium from the proliferative to the secretory phase has significant molecular implications toward enhancing the survival and implantation of the refluxed endometrium. On the one hand, miRNAs have emerged as a major regulatory system of steroid hormone responses in the female reproductive tract (26); on the other hand, several miRNAs are possibly influenced by the hormonal milieu (27,28). The inflammatory environment within the pelvis may also contribute to the pathophysiology of endometriosis. In our study, compared with the disease-free controls, the eutopic endometrium from women with endometriosis showed an increased basal production of IL-6 and TNF-α, which both play a prominent role in a number of chronic inflammatory conditions (29). To determine wether ovarian steroids and inflammatory factors exert regulatory effects on miR-183, we detected miR-183 expression in ESCs treated with 17β-estradiol, progesterone, TNF-α and IL-6 by qPCR. Our results provide evidence that ovarian steroids and inflammatory factors decrease the expression of miR-183 in ESCs; however, further studies are required to examine the molecular mechanisms involved.

In conclusion, we identified several differentially expressed miRNAs, including miR-183 in the normal, eutopic and ectopic endometrium by miRNA microarray screening analysis. The downregulation of the expression of miR-183 enhanced the invasive potential and inhibited the apoptosis of ESCs. The targets of this miRNA related to cellular functions are still being investigated. miR-183 gene expression can be inhibited by the stimulation of ovarian steroids and inflammatory factors; such a regulatory function may further manifest the growth and invasive capacity of ESCs by altering the expression of miR-183. These findings suggest that the aberrant miR-183 expression is involved in the development and progression of endometriosis as part of epigenetic mechanisms.

## Figures and Tables

**Table I:** Differentially expressed miRNAs in ectopic vs. eutopic endometrium and eutopic vs. normal endometrium.

A, Ectopic/eutopic		

miRNAs	Fold-change	P-value
Downregulated		
hsa-miR-203	0.39	0.03
hsa-miR-425	0.25	0.03
hsa-miR-183	0.48	0.01
hsa-miR-92a	0.36	0.03
hsa-miR-196b	0.04	0.03
hsa-miR-363^*^	0.23	0.00
hsa-let-7i	0.32	0.01
hsa-miRPlus-E1031	0.45	0.01
hsa-miRPlus-E1031	0.45	0.01
hsa-miR-200b	0.15	0.05
hsa-miRPlus-F1231	0.30	0.00
hsa-miR-215	0.24	0.01
hsa-miR-362-3p	0.31	0.04
hsa-miR-342-3p	0.27	0.05
hsa-miR-200c	0.17	0.04
hsa-miR-93	0.18	0.01
hsa-miR-24-1^*^	0.45	0.02
hsa-miR-25	0.31	0.03
hsa-miR-106b^*^	0.31	0.00
Upregulated		
hsa-miRPlus-F1038	3.21	0.02
hsa-miR-1915	2.06	0.04
hsa-miR-637	2.35	0.03
hsa-miR-518e^*^	2.22	0.00
hsa-miR-519a^*^		
hsa-miR-519b-5p		
hsa-miR-519c-5p		
hsa-miR-522^*^		
hsa-miR-523^*^		
hsa-miR-574-5p	2.00	0.04
hsa-miR-615-3p	2.14	0.02
hsa-miR-1909	3.17	0.04
hsa-miR-224^*^	2.23	0.02
hsa-miR-133b	2.85	0.03
hsa-miR-622	2.06	0.01
hsa-miR-628-3p	2.27	0.01
ebv-miR-BHRF1-2	2.78	0.03
hsa-miRPlus-F1215	2.28	0.01
hsa-miRPlus-F1221	2.32	0.01
hsa-miR-1470	2.45	0.02
hsa-miR-1469	2.21	0.05
hsa-miR-520d-5p	2.34	0.00
hsa-miR-551b^*^	2.18	0.00
hsa-miR-361-3p	2.83	0.01
hsa-miR-941	3.43	0.03
hsa-miRPlus-F1223	2.50	0.01
hsa-miR-202	12.53	0.01
hsa-miR-663b	2.53	0.03
hsa-miRPlus-F1042	2.33	0.02
hsa-miR-381	2.97	0.01
hsa-miR-412	3.11	0.03

B, Eutopic/normal		

Downregulated		
hsa-miR-921	0.49	0.01
hsa-miR-374b^*^	0.26	0.00
hsa-miRPlus-E1082	0.33	0.03
ebv-miR-BHRF1-3	0.28	0.02
hsa-miR-22^*^	0.41	0.04
hsa-miRPlus-E1027	0.22	0.02
hsa-miR-218-2^*^	0.35	0.02
hsa-miR-148b^*^	0.17	0.00
hsa-miR-183	0.38	0.00
hsa-miRPlus-C1100	0.27	0.02
hsv1-miR-H5-3p	0.40	0.03
hsv1-miR-H4^*^	0.18	0.01
hsa-miR-553	0.35	0.05
hsa-miR-761	0.41	0.04
hsa-miR-302e	0.46	0.04
hsa-miR-9^*^	0.38	0.01
hsa-miRPlus-F1212	0.26	0.03
hsa-miR-363^*^	0.20	0.01
hsa-miR-206	0.23	0.01
hsa-miRPlus-E1192	0.34	0.02
hsa-miRPlus-F1091	0.44	0.04
hsa-miRPlus-E1215	0.31	0.04
hsa-miR-668	0.22	0.00
hsa-miR-215	0.41	0.01
hsa-miR-1265	0.41	0.01
hsa-miR-922	0.24	0.01
hsa-miRPlus-E1185	0.44	0.04
hsa-miRPlus-E1075	0.38	0.02
hsa-miRPlus-E1086	0.39	0.02
hsa-miR-636	0.38	0.02
ebv-miR-BART18-3p	0.24	0.04
hsa-miRPlus-F1074	0.33	0.00
hsa-miRPlus-E1038	0.39	0.00
hsa-miRPlus-E1049	0.30	0.02
hsa-miRPlus-E1193	0.35	0.02
hsa-miRPlus-E1029	0.38	0.01

List of differentially expressed miRNAs whose relative expression value was at least 2-fold higher or lower than the control group (P<0.05).
